# Ionically Crosslinked Chitosan Hydrogels for the Controlled Release of Antimicrobial Essential Oils and Metal Ions for Wound Management Applications

**DOI:** 10.3390/medicines3010008

**Published:** 2016-03-01

**Authors:** Wan Li Low, M.A. (Ken) Kenward, Mohd Cairul Iqbal Mohd Amin, Claire Martin

**Affiliations:** 1 School of Pharmacy Faculty of Science and Engineering, University of Wolverhampton, Wulfruna Street, Wolverhampton, WV1 1LY, UK; w.l.low2@wlv.ac.uk (W.L.L.); m.a.kenward@wlv.ac.uk (M.A.K.); 2 Centre for Drug Delivery Research, Faculty of Pharmacy, Universiti Kebangsaan Malaysia, Jalan Raja Muda Abd Aziz, 50300 Kuala Lumpur, Malaysia; mciamin@pharmacy.ukm.my; 3 Research Institute in Healthcare Science, Faculty of Science and Engineering, University of Wolverhampton, Wulfruna Street, Wolverhampton WV1 1LY, UK

**Keywords:** silver, tea tree oil, hydrogels, chitosan, wound management, antimicrobial activity

## Abstract

The emerging problems posed by antibiotic resistance complicate the treatment regime required for wound infections and are driving the need to develop more effective methods of wound management. There is growing interest in the use of alternative, broad spectrum, pre-antibiotic antimicrobial agents such as essential oils (e.g., tea tree oil, TTO) and metal ions (e.g., silver, Ag^+^). Both TTO and Ag^+^ have broad spectrum antimicrobial activity and act on multiple target sites, hence reducing the likelihood of developing resistance. Combining such agents with responsive, controlled release delivery systems such as hydrogels may enhance microbiocidal activity and promote wound healing. The advantages of using chitosan to formulate the hydrogels include its biocompatible, mucoadhesive and controlled release properties. In this study, hydrogels loaded with TTO and Ag^+^ exhibited antimicrobial activity against *P. aeruginosa*, *S. aureus* and *C. albicans.* Combining TTO and Ag^+^ into the hydrogel further improved antimicrobial activity by lowering the effective concentrations required, respectively. This has obvious advantages for reducing the potential toxic effects on the healthy tissues surrounding the wound. These studies highlight the feasibility of delivering lower effective concentrations of antimicrobial agents such as TTO and Ag^+^ in ionically crosslinked chitosan hydrogels to treat common wound-infecting pathogens.

## 1. Introduction

The increasing occurrence of antibiotic-resistant strains and reports of hospital cross-infection further complicate current practices in wound management. Statistics show that acute and chronic wounds affect approximately 2% of the population, with treatment costs taking up to 4% of the overall health care budget [1]. In Europe, the cost of managing a patient with chronic wounds can cost up to €6000–€10,000 per annum [2].

Acute and chronic wounds require relatively lengthy treatment with antibiotics which carry the attendant risk of developing drug resistance. Additionally, hypersensitivity reactions to antibiotics and the lack of access to new treatments within the health care industry make the care of such patients difficult [3]. This severely compromises the patient’s quality of life and also creates a significant financial burden for the economy. There are, therefore, several forces driving the need to find alternative approaches to conventional wound management.

Management of acute and chronic wounds such as varicose insufficiency ulcers, diabetic foot ulcers and burns aims to minimise infection, speed up wound healing and minimise scarring [4]. Colonisation and subsequent infection of wounds, usually by polymicrobial, opportunistic pathogens, can delay the healing process, and may lead ultimately to potentially fatal, systemic infection [5]. The diversity and proliferation of the pathogens is influenced by various factors including the type, depth and location of the trauma, as well as the host immune system response [5]. The presence of microorganisms at a wound site does not confirm infection [6]. Infection only occurs when the host immune system can no longer cope with the virulence factors expressed by the colonizing microorganisms, thus triggering a series of systemic responses which delay the healing process [5,6]. Topical application of antimicrobial agents is a popular approach to wound management since effective concentrations may be difficult to achieve with systemically delivered drugs [3,7].

The problems posed by increasing antibiotic resistance in Gram-positive (e.g., Methicillin Resistant *Staphylococcus aureus*, (MRSA)) and Vancomycin-resistant enterococci) and Gram-negative bacteria (e.g., New Delhi metallo-β-lactamase-1 and ciprofloxacin-resistant *Pseudomonas aeruginosa*) [8,9] have renewed interest in pre-antibiotic antibacterial agents such as essential oils and metal ions [10,11].

The essential oil tea tree oil (TTO) contains >100 different components which contribute to its broad spectrum antibacterial, antifungal, antiviral, antimycoplasmal, antiprotozoal and anti-inflammatory activity [12]. Commercially available TTO contains mainly monoterpenes, approximately 50% of which are oxygenated [13] sesquiterpenes and their associated alcohols [12]. The major component of TTO, terpinen-4-ol, is primarily responsible for its antimicrobial activity [12]. The hydrophilic hydrocarbon compounds in TTO have sufficient lipophilicity to allow the oil to partition preferentially into biological membranes resulting in bilayer expansion [12]. TTO components can easily diffuse through the hydrophobic lipid bilayer of the microbial cell membrane, causing disruption to integrity and function, increased fluidity, loss of permeability and inhibition of embedded membrane enzymes. Consequently, the cell loses essential metabolites and repair enzymes, ultimately resulting in cell death [12,13]. The microbiocidal properties of active monoterpenes are mainly attributed to disruption of the cell membrane’s barrier function; cells are thus unable to establish control over membrane-coupled energy-transducing processes, solute transport, regulation of metabolism and maintenance of turgor pressure [13]. Preparations containing TTO are commonly used for their antiseptic, antimicrobial, cleansing, healing and itch-relieving properties [14]. In addition, TTO is widely used in wound management for its antimicrobial and therapeutic properties: e.g., Burnaid^®^ (Rye Pharmaceuticals, Roseville, NSW, Australia) is a commercial hydrogel dressing impregnated with TTO for the treatment of burns [15]. The topical application of TTO is not without its problems, however; there are reports of dermal toxicity of TTO resulting in irritation and allergic reactions [12]. The use of TTO-based dressings on large areas of the skin whilst providing a desirable localized cooling effect on burns may trigger an unwanted hypothermic response [15].

Silver ions (Ag^+^) have regained popularity as an antimicrobial agent due to their broad spectrum antibacterial, antiviral, antiprotozoal and antifungal activity [16]. Ag^+^ are classified as highly reactive moieties, which readily bind anions formed by electron donor groups containing sulphur (thiols), oxygen and nitrogen [16]. The mode of action involves the inactivation of membrane-bound proteins, resulting in morphological cellular changes, inhibition of cell replication [17,18] and impairment of solute and electron transport systems. This interferes with the activity of essential intracellular enzymes and DNA, leading to reduced production of vital cell components, such as adenosine triphosphate (ATP) [16,18,19,20]. The ability of Ag^+^ to affect multi-target sites is beneficial to reduce the potential development of resistant strains [21]. In addition, Ag^+^ has been reported to enhance wound healing directly by modulating the inflammatory response [22]. The antimicrobial activity and inflammatory regulation activities of Ag^+^ are of interest in the development of improved wound management products. Formulations containing silver are commonly used to treat a variety of Gram-positive and Gram-negative bacteria, as well as common highly-antibiotic-resistant microorganisms such as *P. aeruginosa* [18]. Silver-based pharmaceutical preparations, e.g., silver sulfadiazine (Flamazine^®^, Smith and Nephew Healthcare Limited, Hull, UK), have been successfully used for the treatment of burn wound infections. In addition, controlled release delivery systems have also incorporated Ag^+^ to reduce infection and improve the wound healing process. Some current examples of silver-containing wound dressings include DuoDERM^®^ (hydrocolloids, ConvaTec, Skillman, NJ, USA), Aquacel^®^ Ag (hydrofibre dressings containing antimicrobial Ag^+^, ConvaTec, Skillman, NJ, USA), Tegaderm^TM^ (films, 3M United Kingdom PLC, Bracknell, UK), Vaseline^TM^ (gauze, Unilever, London, UK), Sorbsan^®^ (alginates, Bertel Pharmacceuticals, Research Triangle Park, NC, USA), Lyofoam^®^ C (foam dressing, ConvaTec, Skillman, NJ, USA) and Nu-Gel^TM^ (hydrogels, Johnson and Johnson Wound Management, Somerville, NJ, USA) [23,24]. As is common with many drug treatments, over-exposure to Ag^+^ can trigger unwelcome side effects. Long-term topical exposure to high concentrations of Ag^+^ leads to a build-up of metallic silver (Ag^0^) in the dermis, causing an irreversible blue-grey discolouration of the skin (argyria). This is particularly pronounced in areas exposed to sunlight which accelerates the photo-reduction and deposition of Ag^0^ [25,26,27]. Some patients treated with silver-containing dressings reported the occurrence of skin rashes, stinging and burning sensations [28]. Other more serious problems associated with the topical application of silver include disturbances in electrolyte concentration resulting in hyponatremia or hypochloremia [20].

Hydrogels are three-dimensional crosslinked polymer networks which can imbibe large amounts of water or biological fluids [4,29]. These biodegradable gels maintain their structure during the exchange of fluids and provide controlled release of drugs *via* pores in the polymer network [29,30]. When applied to wounds, hydrogels can absorb exudate while maintaining a well-oxygenated and moist environment [31,32]. The parameter that dictates hydrogel formation is the constituent polymer’s aqueous solubility, whether it is from natural or synthetic sources. Chitosan, a natural polymer amenable to hydrogel formation, is a linear copolymer of β(1-4)-linked 2-acetamido-2-deoxy-d-glucopyranose and 2-amino-2-deoxy-d-glyco-pyranose monomers [32]. Chitosan has good biocompatibility, biodegradability, mucoadhesive and low toxicity properties. It forms a barrier to gases and aromas in dry conditions, and is used to prepare membranes, films, microparticles, nanofibrils, scaffolds and gels [33,34,35]. Chitosan’s properties can be adapted to suit a wide variety of antimicrobial delivery systems for medical, food, coating and packaging applications. In addition, chitosan has bacteriostatic properties against fungi and Gram-negative bacteria. The advantages of using chitosan for wound management applications are its biocompatible, mucoadhesive and controlled release properties. This allows cell adhesion and interactions which promote the growth of dermal and epidermal layers by inducing cell migration as well as proliferation [35,36].

The useful properties of alternative antimicrobial agents, together with advances in drug delivery technologies, may be able to enhance and expand the medical applications of these agents. Combining these alternative antimicrobial agents with advanced drug delivery systems aims to: (1) promote bioavailability of the agent at microbiocidal concentrations; (2) reduce drug concentration to enhance safety and practicality of application; (3) minimise scarring and promote wound healing processes; (4) reduce discomfort and pain in consideration of the patients’ psychological needs; and (5) decrease the frequency of dressing changes [37]. These aims would increase convenience, provide less opportunity for infection and/or reinfection of the wound and ultimately reduce treatment costs.

Therefore, the aims of this investigation were to examine the *in vitro* antimicrobial efficacy of hydrogels incorporating TTO alone and in combination with Ag^+^ (as silver nitrate, AgNO_3_) against representative wound-infecting organisms, namely *P. aeruginosa*, *S. aureus* and *C. albicans*. We report here on the characterisation and optimisation of both preparation and formulation techniques of a hydrogel-based delivery system for controlled release of antimicrobially active Ag^+^ and TTO.

## 2. Materials and Methods

### 2.1. Preparation of Microbial Cultures

Sterile tryptone soy broth (TSB), tryptone soy agar (TSA), malt extract broth (MEB) and malt extract agar (MEA) were obtained from Lab M, Lancashire, UK, and prepared according to manufacturer’s recommendations. Overnight culture of *P. aeruginosa* (NCIB 8295) was prepared by aseptically inoculating 50 mL of sterile TSB and incubating overnight in an orbital shaker at 37 °C. Similarly, overnight cultures of *S. aureus* (NCIB 6571) in TSB and *C. albicans* (NCYC 854) in MEB were also similarly prepared.

### 2.2. Hydrogel Formulation and Characterisation

Sodium phosphate (monobasic monohydrate, Sigma Aldrich, Darmstadt, Germany), Na-P solutions (25% and 50% *w*/*v*) were prepared in sterile distilled water. Medium- or low-viscosity chitosan (Sigma Aldrich, Darmstadt, Germany) solutions (1.25% and 2.5% *w*/*v*) were prepared in 1.0% *v*/*v* glacial acetic acid (BDH, Poole, UK). Phosphate buffer saline (PBS) (Invitrogen Corporation, Camarillo, CA, USA) was prepared according to manufacturer’s recommendation and autoclaved prior to use.

Ionically crosslinked chitosan hydrogels were prepared by vigorous magnetic stirring of various ratios of chitosan (medium or low viscosity, 1.25% or 2.5% *w*/*v*), Na-P (25% or 50% *w*/*v*) and 10% *w*/*v* poly(vinyl alcohol) (PVA) 13–23 kDa, PVA_13-23_, or 31-50kDa, PVA_31-50_, (Sigma Aldrich, Darmstadt, Germany) solutions. After mixing for 2 min the gelling mixtures were passed through 0.45 μm sterile syringes, transferred into sterile Petri dishes, covered and allowed to set for a minimum of 2 h.

The physical stability of each formulation was assessed firstly by confirming the formation of a uniform hydrogel. Following that, discs of hydrogel (diameter 8 mm) were cut and placed in a clean Petri dish with 20 mL of distilled water. Hydrogel formulation discs that maintained their shape, could easily be cut out and handled, as well as remaining stable after incubation in water were chosen for further study.

TTO:PVA (poly[vinyl alcohol]) emulsions were prepared by probe sonicating (Bandelin Sonopuls HD2200, Bandelin Electronic, Berlin, Germany) TTO and 10% *w*/*v* PVA (35:65 *v*/*v* ratio) for 1 min. TTO:PVA-loaded hydrogels were prepared by substituting or adding sufficient emulsified TTO as part of the PVA component in the formulations to produce hydrogels with final TTO concentration of 5% or 7% *v*/*v*.

TTO-encapsulated hydrogel discs (8 mm diameter) were incubated in 20 mL PBS at room temperature. At time zero and at every hour up to 6 h, 6 mL of PBS was sampled, and replaced by 6 mL of fresh PBS. Further samples were taken after 7.5 and 24 h. Subsequently the amount of TTO and chitosan released from the hydrogels was quantified using the monoterpene and ninhydrin quantification assays, respectively.

### 2.3. Vanillin Assay for the Determination of Monoterpene Release from TTO

A colorimetric method for determining the total monoterpene content using 2.5% *w*/*v* vanillin (ReagentPlus^®^ grade, Acros Organics, Geel, Belgium) in concentrated sulphuric acid (Philip Harris Scientific, Lichfield, UK) was adapted from Doneva-Šapceska *et al.*, 2006 [38]. Vanillin reagent (2.5 mL) was added to test tubes containing 5 mL of sample, mixed and heated in a 60 °C water bath for 20 min. The samples were then cooled in an ice-water bath to 25 °C. A blank sample was also prepared using 5 mL distilled water. Absorbance of the samples was measured spectrophotometrically at 608 nm (Cecil 1000 Series, Cecil Instruments, Cambridge, UK).

### 2.4. Chitosan Assay (Ninhydrin Assay)

Quantification of chitosan was performed using a colorimetric ninhydrin assay adapted from Leane *et al.*, 2004 [39]. Lithium acetate buffer (4 M) was prepared by dissolving 4.08 g of lithium acetate dihydrate (Aldrich grade 98%, Sigma Aldrich, Milwaukee, WI, USA) in 10 mL distilled water and adjusted to pH 5.2 using glacial acetic acid. Ninhydrin reagent was prepared by adding 0.8 g ninhydrin (ACS reagent, Sigma Aldrich, Bangalore, India) and 0.12 g hydrindantin (Sigma Aldrich, Vienna, Austria) in 30 mL dimethylsulphoxide (DMSO) (Calbiochem, EMD Chemicals, San Diego, CA, USA) into 10 mL of 4 M lithium acetate buffer. Ninhydrin reagent (0.5 mL) was added to 0.5 mL of sample in a capped test tube. Blanks sample were prepared by substituting the sample with distilled water. The contents were mixed and heated in boiling water for 30 min, cooled and 15 mL of 50:50 ethanol:water cosolvent mixture added. The contents of each tube were vortexed for 15 s to ensure complete oxidization of the excess hydrindantin, prior to measuring absorbance spectrophotometrically at 570 nm (Cecil 1000 Series). The results were analysed by fitting the data to zero order, first order, Higuichi and Korsmeyers-Peppas equations [40] to deduce the monoterpene release profile from the hydrogels.

### 2.5. Formulation of Ag^+^ and TTO:Ag^+^:PVA for Antimicrobial Studies

Following the initial trial formulations confirming the feasibility of hydrogels to deliver TTO, another batch of hydrogels containing 35% TTO: 65% PVA_30-70_ *v*/*v*, Ag^+^ and TTO:Ag^+^:PVA_30-70_ were produced. It was found that the Ag^+^ solutions used in the formulations also acted as the crosslinker for the hydrogels; hence, in the formulations containing Ag^+^, Na-P was substituted with 1% or 2% *w*/*v* AgNO_3_. The emulsion of TTO:Ag^+^:PVA_30-70_ was prepared by dissolving sufficient AgNO_3_ in 10% *w*/*v* PVA_30-70_, adding sufficient TTO and sonicating the mixture for 3 min. The concentration of TTO:Ag^+^:PVA_30-70_ used were as follows:

- 40% TTO: 0.5% AgNO_3_
- 20% TTO: 1.0% AgNO_3_

Table 1 shows the detailed composition of stable hydrogels containing TTO and/or Ag^+^, which were used for antimicrobial studies.

### 2.6. Well Diffusion Assay to Determine the Antimicrobial Activity of Hydrogels

The antimicrobial effect of the agents encapsulated into hydrogels was tested using a standard well diffusion method. Overnight cultures of *P. aeruginosa* and *S. aureus* were adjusted accordingly to the following absorbance reading at 500 nm based on the British Society for Antimicrobial Chemotherapy (BSAC) disc diffusion method guidelines [41]:

- *P. aeruginosa* −> 0.1−0.3
- *S. aureus* −> 0.3−0.6

Overnight cultures of *C. albicans* were used without dilution in the disc diffusion experiments, as they contained sufficient number of cells for the experiments. The adjusted cultures were swabbed on the surface of the agar plates in triplicate according to the BSAC disc diffusion method recommendations. A well (12 mm diameter) was aseptically bored in the middle of the agar plate using a sterile metal borer. The hydrogel was aseptically placed in the well and incubated overnight at 37 °C, prior to measuring the diameter of the zone of inhibition. Control experiments were prepared by substituting the hydrogels with those formulated without TTO:PVA and AgNO_3_.

## 3. Results and Discussion

### 3.1. Appearance of Ionically Crosslinked Chitosan Hydrogels

**Figure 1:**
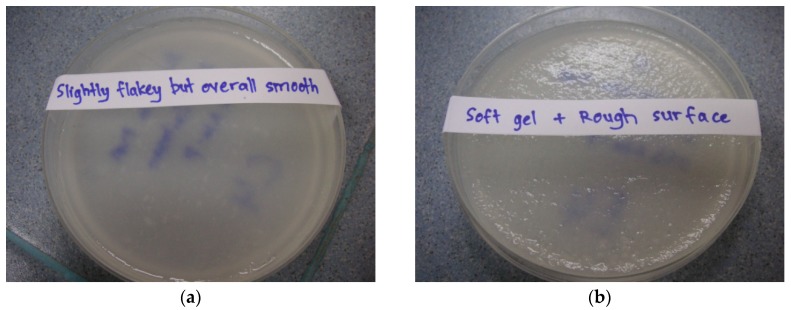

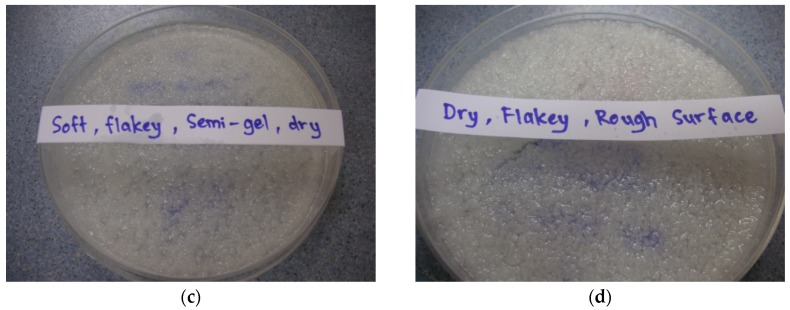
Appearance of ionically crosslinked hydrogels. (**a**): Hydrogels with a slightly flakey but overall smooth surface; (**b**) Hydrogels with soft texture and rough surface; (**c**) Semi-gel, soft hydrogels with flakey and dry surface ; (**d**) Hydrogels with dry, flakey, rough surface.

Data from the preliminary studies showed that stable, uniform hydrogels were formed using the composition ratios as detailed in Table 2. All the hydrogel formulations reported in Table 2 could easily be cut out as 8 mm or 12 mm diameter discs, remained stable when incubated in PBS and did not break up extensively over the incubation period. Examples of the appearance of the formulated hydrogels are shown in Figure 1.

### 3.2. Cumulative Chitosan and Monoterpene Release after 24 h Incubation

**Figure 2:**
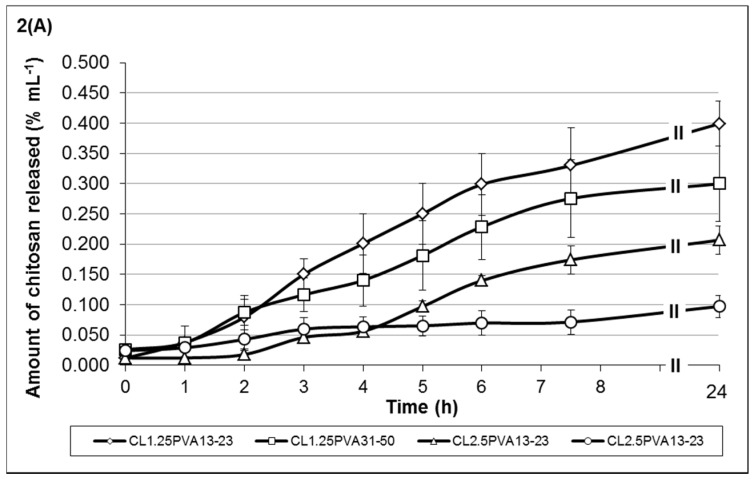

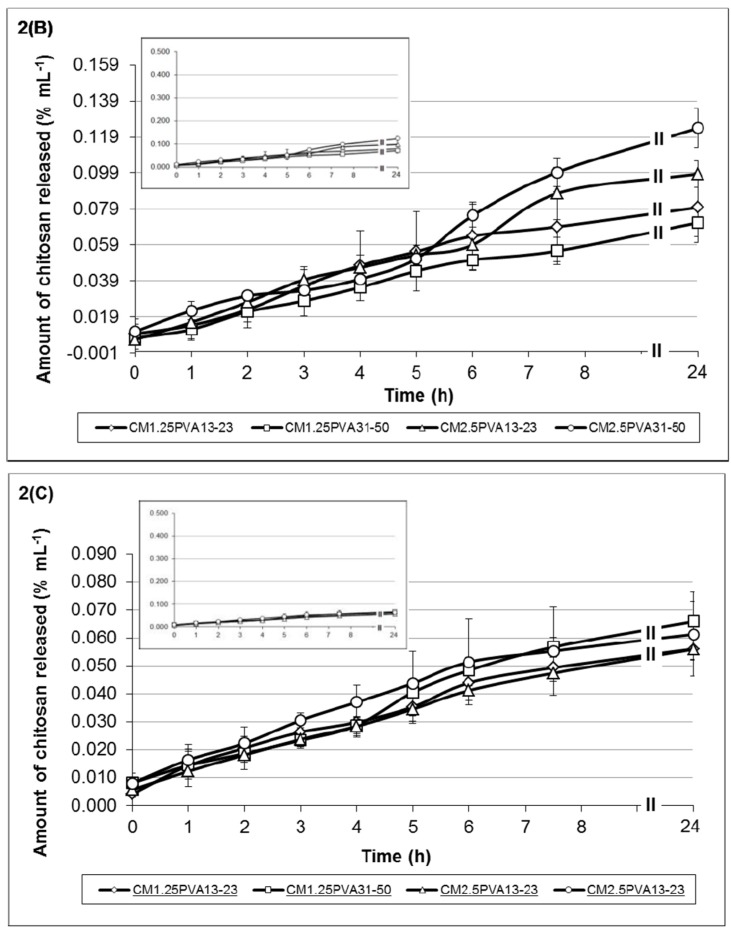
Cumulative release of (**A**) low-viscosity chitosan; and (**B**) medium-viscosity chitosan from hydrogel formulations containing TTO: 10 *w*/*v* PVA (35:65 *v*/*v* ratio); and (**C**) medium-viscosity chitosan from hydrogel formulations containing TTO: 10 *w*/*v* PVA (50:50 *v*/*v* ratio). A sub-graph has been provided in both (**B**, **C**) to provide an overview of the amount of released chitosan in comparison to (2**A**). The codes shown in the figure key describe the following: C_L1.25_ = 1.25% *w*/*v* low-viscosity chitosan, C_L2.5_ = 2.5% *w*/*v* low-viscosity chitosan, C_M1.25_ = 1.25% *w*/*v* medium-viscosity chitosan, C_M2.5_ = 2.5% *w*/*v* medium-viscosity chitosan, PVA_13-23_ = PVA 13–23 kDa, PVA_31-50_ = PVA 31–50 kDa. The error bars represents the standard deviation and *n* = 3.

**Figure 3:**
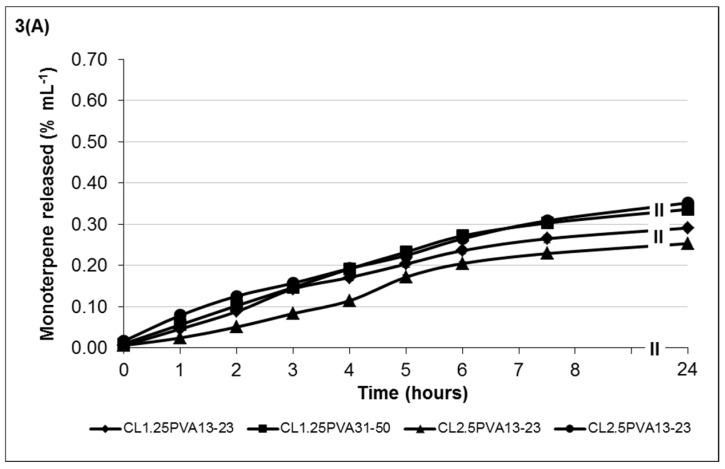

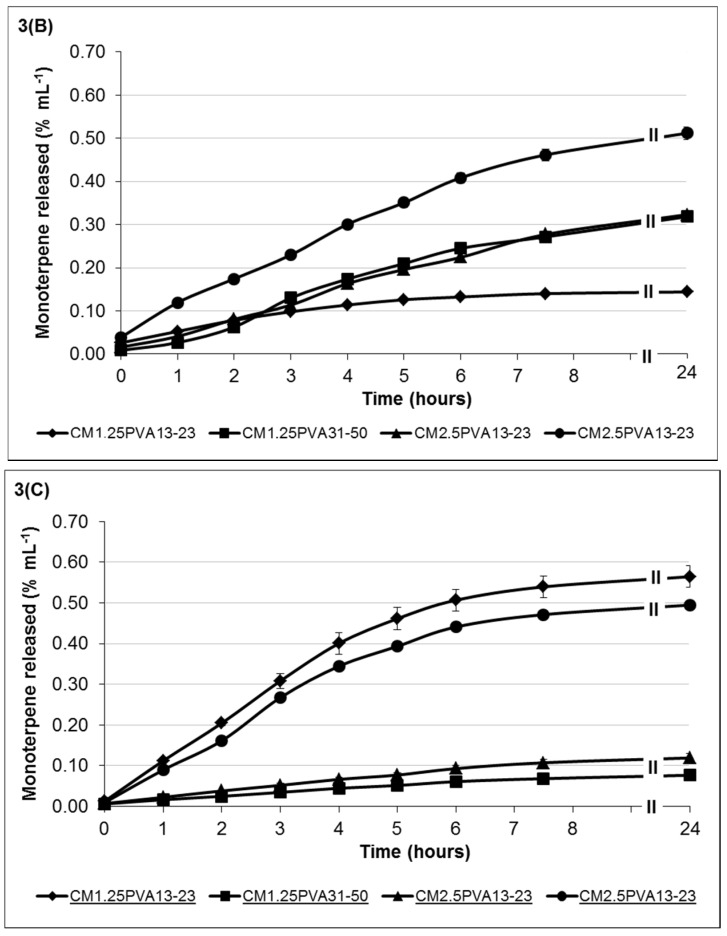
Cumulative release of monoterpene from (**A**) low-viscosity chitosan; and (**B**) medium-viscosity chitosan hydrogel formulations containing TTO: 10% *w*/*v* PVA (35:65 *v*/*v* ratio); and (**C**) medium-viscosity chitosan hydrogel formulations containing TTO: 10% *w*/*v* PVA (50:50 *v*/*v* ratio). The codes shown in the figure key describes the following: C_L1.25_ = 1.25% *w*/*v* low-viscosity chitosan, C_L2.5_ = 2.5% *w*/*v* low-viscosity chitosan, C_M1.25_ = 1.25% *w*/*v* medium-viscosity chitosan, C_M2.5_ = 2.5% *w*/*v* medium-viscosity chitosan, PVA_13-23_ = PVA 13–23 kDa, PVA_31-50_ = PVA 31–50 kDa. The error bars represents the standard deviation and *n* = 3.

The release mechanism of chitosan and monoterpene (TTO) from hydrogels was investigated as a function of hydrogel degradation. Figure 2 shows the release profiles of low-viscosity chitosan (Figure 2A) and medium-viscosity chitosan (Figure 2B) of hydrogel formulations containing 35% TTO: 65% PVA (10% *w*/*v*) emulsion. The release profiles of chitosan from formulations containing 50% TTO: 50% PVA (10% *w*/*v*) are shown in Figure 2C. Similarly, the monoterpene release profiles from the respective hydrogel formulations are shown in Figure 3.

Low-viscosity chitosan formulations showed chitosan release was between 9.72 × 10^−2^ to 3.99 × 10^−1^ %/mL at 24 h post-incubation (Figure 2A). Comparatively, the amount of monoterpene release was fairly similar, *i.e.*, 2.53 × 10^−1^ to 3.52 × 10^−1^ %/mL (Figure 3A). Results showed that lower-viscosity hydrogels and those formulated using PVA_13-23_ (to emulsify the TTO prior to encapsulation) eroded at a higher rate (more chitosan released). The hydrogels formulated with PVA_13-23_ and chitosan_L1.25_ released 3.99 × 10^−1^ %/mL compared to 2.07 × 10^−1^ %/mL in gel formulated using chitosan_L2.5_. Similarly, in hydrogels formulated with PVA_31-50_, stability was also improved in gels formulated with chitosan_L2.5_, resulting in 9.72 × 10^−2^ %/mL chitosan release compared to 2.99 × 10^−1^ %/mL in the gel formulated using chitosan_L1.25_.

The trend of chitosan and monoterpene release from hydrogels formulated with medium-viscosity chitosan differs from the low-viscosity hydrogels. Results in Figure 2B and Figure 3B showed that chitosan_M1.25_ formulations gradually released both chitosan and monoterpene over time. The formulation having a higher volume of 25% *w*/*v* Na-P (formulation C_M1.25_PVA_13-23_) displayed better stability properties, *i.e.*, low chitosan release (low erosion) with slow release of monoterpene. In contrast, formulations with chitosan_M2.5_ showed a steep increase in chitosan release between 5 and 7.5 h post-incubation, resulting in the higher final amount released. Although the gels formulated with chitosan_M2.5_ are considered to be more stable, those formulated with PVA_13-23_ (formulation C_M2.5_PVA_13-23_) may be more prone to erosion due to the lower chitosan:Na-P ratio, resulting in weaker crosslinking. Similarly, the stability of the gels may also be compromised by the chitosan-PVA chain entanglement interaction. Hence, despite the higher chitosan:Na-P ratio, the gels formulated with PVA_31-50_ (formulation C_M2.5_PVA_31-50_) appear to be less stable (greater chitosan erosion) due to the dissociation of the chitosan-PVA interaction. Consequently, this also resulted in higher monoterpene release, despite the more viscous properties of these formulations within the group.

When formulating medium-viscosity hydrogels with 50% TTO:50% PVA *v*/*v*, there were observable changes in the chitosan and monoterpene release profiles (Figure 2C and Figure 3C). The overall chitosan release from the formulations was lower when compared to the formulations in Figure 2B. All formulations showed gradual increase of chitosan release over time, except formulation C_M1.25_PVA_31-50_ in Figure 2C, which showed a steep increase between 4 and 6 h post-incubation.

Similar to the other formulations, increasing crosslinking along with increased viscosity improves hydrogel stability. These gels (C_M1.25_PVA_13-23_ and C_M2.5_PVA_31-50_ in Figure 3C) showed a gradual increase in monoterpene release up to 5.65 × 10^−1^ %/mL and 4.95 × 10^−1^ %/mL, respectively. On the other hand, monoterpene release in the formulations having a lower ratio of chitosan:Na-P (less viscous) was between 7.65 × 10^−2^ and 1.19 × 10^−1^ %/mL. When monoterpene release for hydrogel formulations was analysed with zero order, first order, Higuchi and Korsmeyer-Peppas equations [40], the results indicated that monoterpene release from the all the hydrogel formulations, except formulation C_M1.25_PVA_13-23_, follows the zero order release with a correlation coefficient of R > 0.97, *i.e.*, release of monoterpene was constant over time [40]. The correlation coefficients for the other models (first order release, Korsmeyers-Peppas and Higuchi models) were all <0.97 (Table 3). Based on the mathematical modelling, the release profile of formulation C_M1.25_PVA_13-23_ fits the Higuichi equation (R^2^ > 0.97); hence, the monoterpene was released via a diffusional process according to Fick’s law [40].

Preferably, TTO-hydrogel formulations should be tailored to have lower chitosan release along with more controlled monoterpene release, thus indicating increased formulation stability with sustained delivery of TTO to maintain antimicrobial efficacy. It is evident that there are many factors which may influence the viscosity and properties of each formulation.

### 3.3. Well Diffusion Assay of Hydrogels Containing TTO and/or Ag^+^

The results from the well diffusion assay showed antimicrobial activity in the presence of the agents both singly and in combination. Increasing the viscosity of chitosan from low to high effectively increases the respective molecular weight and polymeric chain length [42], which results in potentially greater inter-chain entanglement, hence affecting its viscosity and ability to release the antimicrobial agents. The antimicrobial activity of the formulations will be discussed separately based on the type of agent and chitosan viscosity.

**Figure 4:**
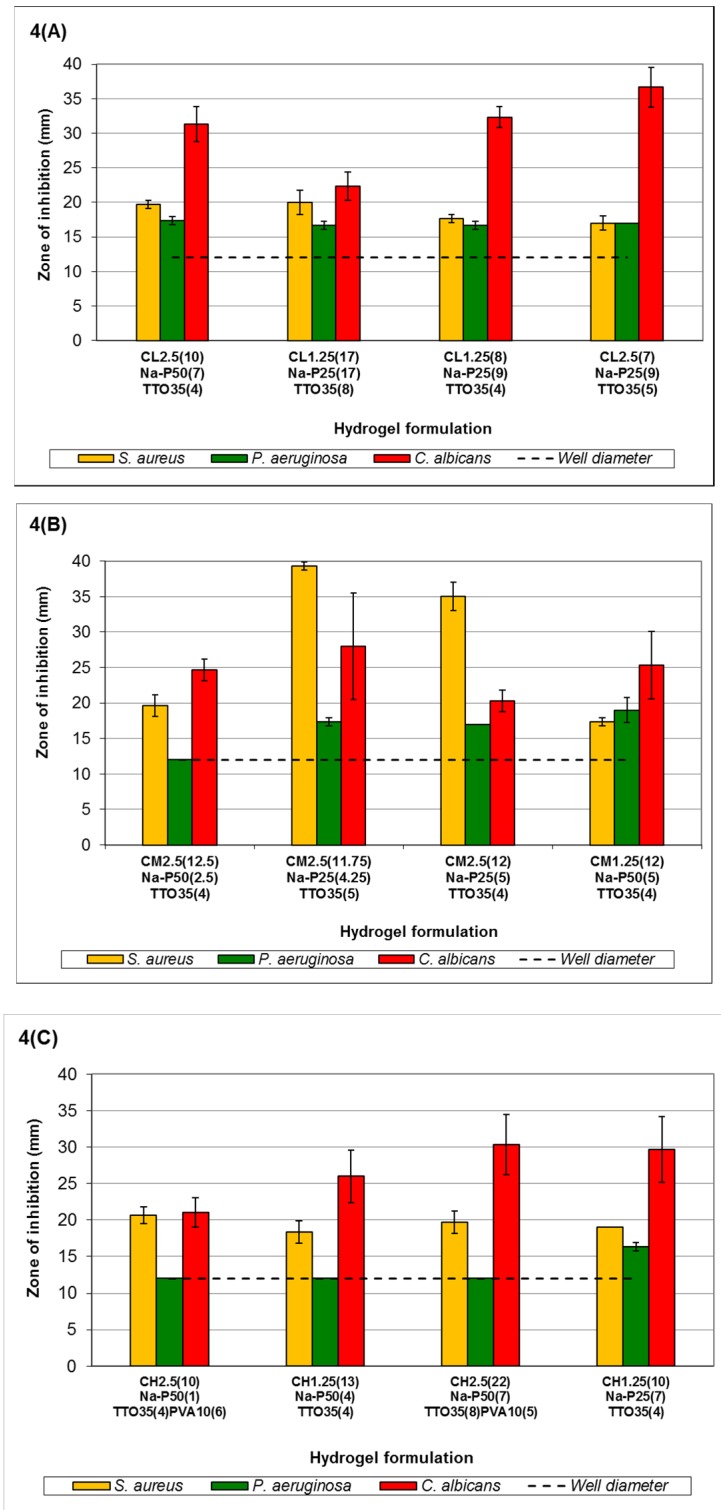
Antimicrobial activity of TTO-hydrogels formulated with (**A**) low viscosity chitosan, (**B**) medium viscosity chitosan and (**C**) high viscosity chitosan. Error bars represent the standard deviation and *n* = 3. In all control experiments (using hydrogels without TTO), there was no observed zone of inhibition (ZOI) (data not shown). Statistical analysis using ANOVA indicated a significant difference in the antimicrobial activity of the formulations against the selected microorganisms (*p* < 0.05). The codes shown in the figure key describes the following: C_L1.25_ = 1.25% *w*/*v* low viscosity chitosan, C_L2.5_ = 2.5% *w*/*v* low viscosity chitosan, C_M1.25_ = 1.25% *w*/*v* medium viscosity chitosan, C_M2.5_ = 2.5% *w*/*v* medium viscosity chitosan, C_H1.25_ = 1.25% *w*/*v* high viscosity chitosan, C_H2.5_ = 2.5% *w*/*v* high viscosity chitosan, Na-P_25_ = 25% *w*/*v* sodium phosphate, Na-P_50_ = 50% *w*/*v* sodium phosphate, TTO_35_ = emulsion of TTO:PVA_30-70_ (35:65 *v*/*v* ratio), PVA = PVA 30-70kDa and the volume of the components used in each formulation are shown in the brackets.

The effect of TTO-hydrogel (low viscosity) formulations follows an order of sensitivity similar to that of the minimum lethal concentration (MLC) reported in Low *et al.* (2011) [43] for free TTO, *i.e.*, *C. albicans* > *S. aureus* ≥ *P. aeruginosa* (Figure 4A). Results showed little changes in the zones of inhibition (ZOI) against *P. aeruginosa*, possibly due to the higher tolerance of *P. aeruginosa* to TTO. In Figure 4A, the formulations C_L1.25(17)_Na-P_25(17)_TTO_35(8)_-C_L2.5(7)_Na-P_25(9)_TTO_35(5)_ vary with increasing viscosity and amount of crosslinker which can improve gel stability. This resulted in more stable TTO hydrogels which can entrap more of the essential oil; the improved antimicrobial activity is observed by an increased ZOI for microorganisms with higher TTO sensitivity, e.g., *C. albicans*. Despite increased formulation stability (C_L1.25(17)_Na-P_25(17)_TTO_35(8)_-C_L2.5(7)_Na-P_25(9)_TTO_35(5)_), the ZOI for less sensitive microorganisms, e.g., *P. aeruginosa* and *S. aureus*, has not been significantly affected (*p* > 0.05). This may be due to the slower rate of TTO release (from more stable hydrogels) which limits the availability of the agent at microbiocidal concentrations, and thus the ZOI for less sensitive microorganisms are not affected. Conversely, formulation C_L2.5(10)_Na-P_50(7)_TTO_35(4)_ had the lowest chitosan: Na-P ratio which makes it less stable and more prone to faster erosion and drug release, resulting in a burst release of TTO which leads to a higher ZOI after 24 h incubation. Such immediate burst release with concomitant rapid hydrogel erosion is not particularly useful when treating wounds with heavy microbial loads. Formulations therefore need to be optimised to reduce the rate of erosion and degradation of the hydrogel whilst maintaining prolonged delivery of agents at microbiocidal concentrations.

Results from Figure 4B did not show significant ZOI changes with increasing stability (C_M2.5(11.75)_Na-P_25(4.25)_TTO_35(5)_-C_M1.25(12)_Na-P_50(5)_TTO_35(4)_) against *P. aeruginosa*. The ZOI in hydrogels C_M2.5(12.5)_Na-P_50(2.5)_TTO_35(4)_ and C_M1.25(12)_Na-P_50(5)_TTO_35(4)_ follows the MLC sensitivity trend of *C. albicans* > *S. aureus* > *P. aeruginosa*. However, the extent of antimicrobial activity, especially against the more sensitive *C. albicans*, is lower than those formulated with low-viscosity chitosan (C_L2.5(10)_Na-P_50(7)_TTO_35(4)_-C_L2.5(7)_Na-P_25(9)_TTO_35(5)_). This reduce in antimicrobial activity is probably due to the overall increase in chitosan chain length (moving from low to medium viscosity), which potentially increases inter-chain entanglement, hence retarding the ability of TTO diffusion and thereby reducing the amount released from the hydrogel structure. Within this group, formulation C_M2.5(12.5)_Na-P_50(2.5)_TTO_35(4)_ (lowest chitosan:Na-P ratio) forms a less stable hydrogel with a higher rate of erosion; the reduced crosslinking also contributes to a less stable gel network to maximise TTO:PVA encapsulation by allowing chain entanglement interactions between PVA and chitosan. As a result, TTO escape from the hydrogel may again follow a burst release profile and increase the ZOI for *C. albicans* and *S. aureus*. However, the concentration was insufficient to show a clear ZOI for *P. aeruginosa*, although there was an observed zone of restricted growth with an average diameter of 30.33 mm. Formulations C_M2.5(12)_Na-P_25(5)_TTO_35(4)_ and C_M1.25(12)_Na-P_50(5)_TTO_35(4)_ are more stable due to their increased viscosity (there is more chitosan in the formulation which is not diluted by the addition of aqueous Na-P). Despite having the same chitosan:Na-P ratio, the antimicrobial performance of C_M2.5(12)_Na-P_25(5)_TTO_35(4)_ and C_M1.25(12)_Na-P_50(5)_TTO_35(4)_ was different. This may be due to the variations in Na-P concentration, which can alter crosslinking and viscosity of hydrogels (C_M2.5(12)_Na-P_25(5)_TTO_35(4)_ formulated using 25% *w*/*v* Na-P, C_M1.25(12)_Na-P_50(5)_TTO_35(4)_ formulated using 50% Na-P). Such properties include both the viscosity of the various chitosan solutions and the ability of oppositely charged crosslinking phosphate anions to diffuse into the solution and form an electrostatically crosslinked semi-solid structure. The order of sensitivity in formulation C_M2.5(11.75)_Na-P_25(4.25)_TTO_35(5)_ was altered to *S. aureus* > *C. albicans* > *P. aeruginosa*, which could have resulted from the higher concentration of TTO present in the hydrogels.

Figure 4C showed that antimicrobial activity of hydrogels formulated with high-viscosity chitosan against *C. albicans* increased with increasing viscosity. The ZOI against *S. aureus* remained fairly consistent, possibly due to the increased gel stability and higher encapsulation of TTO and its components, which are subsequently available for release. Formulation C_H2.5(10)_Na-P_50(1)_TTO_35(4)_PVA_10(6)_-C_H2.5(22)_Na-P_50(7)_TTO_35(8)_PVA_10(5)_ showed no clear ZOI against *P. aeruginosa*; however, similar to formulation C_M1.25(12)_Na-P_50(5)_TTO_35(4)_, there was an insufficient concentration of TTO to result in a clear ZOI and zones of restricted growth with average diameters of 13.5 mm (C_H2.5(10)_Na-P_50(1)_TTO_35(4)_PVA_10(6)_), 33.0 mm (C_H1.25(13)_Na-P_50(4)_TTO_35(4)_) and 30.667 mm (C_H2.5(22)_Na-P_50(7)_TTO_35(8)_PVA_10(5)_) were observed. Formulation C_H1.25(10)_Na-P_25(7)_TTO_35(4)_ was shown to have a ZOI against *P. aeruginosa*. The results in Figure 4 indicated that changes in hydrogel composition can affect their respective antimicrobial activities, possibly due to alterations in physicochemical properties of the gels.

**Figure 5:**
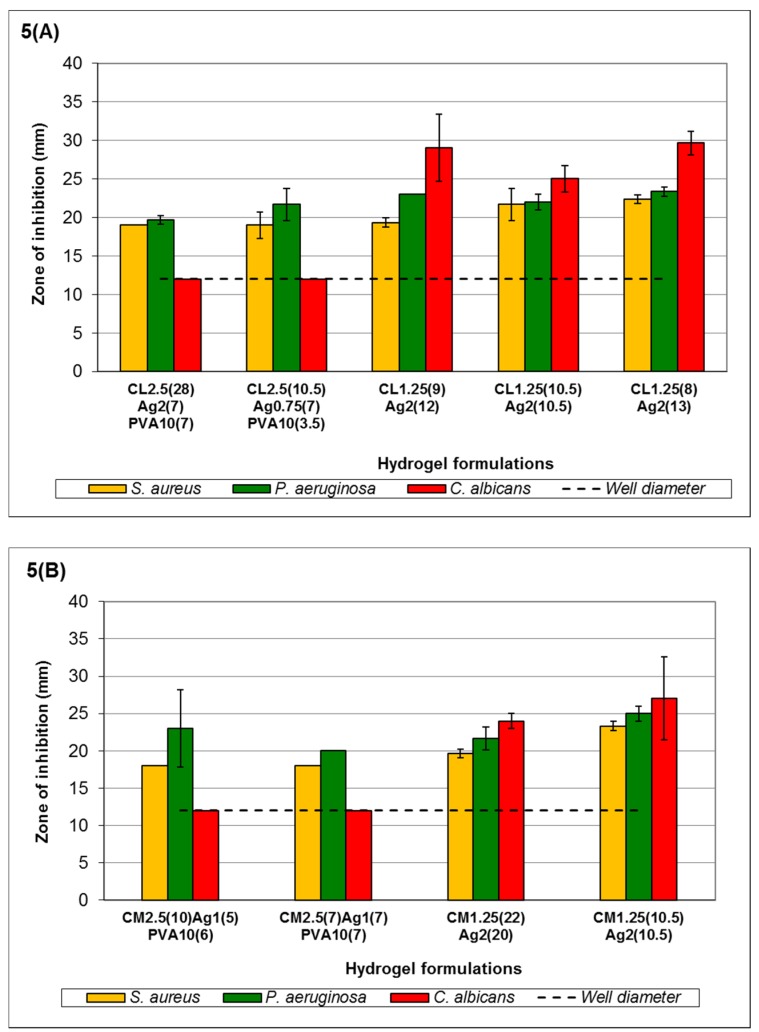

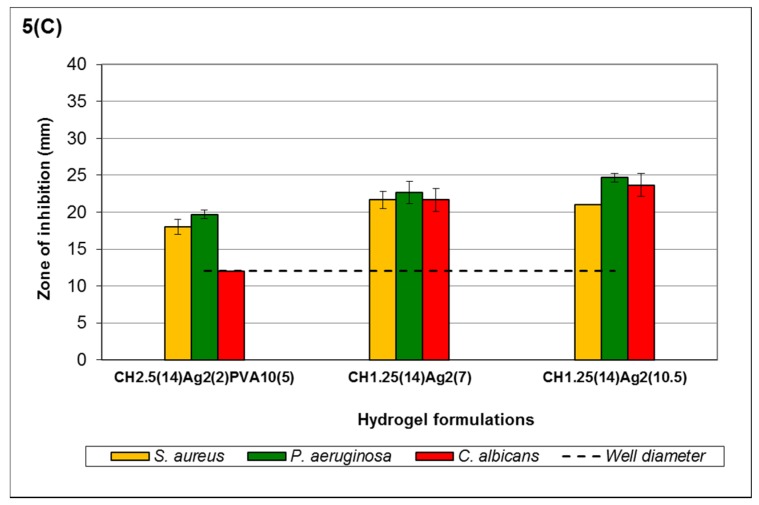
Antimicrobial activity of Ag^+^ hydrogels formulated with (**A**) low-viscosity chitosan; (**B**) medium-viscosity chitosan; and (**C**) high-viscosity chitosan. Error bars represent the standard deviation and *n* = 3. In all control experiments (using hydrogels without Ag^+^), there was no observed ZOI (data not shown). Statistical analysis using ANOVA indicated no significant difference in the antimicrobial activity of the formulations against the selected microorganisms (*p* > 0.05). The codes shown in the figure key describe the following: C_L1.25_ = 1.25% *w*/*v* low-viscosity chitosan, C_L2.5_ = 2.5% *w*/*v* low viscosity chitosan, C_M1.25_ = 1.25% *w*/*v* medium viscosity chitosan, C_M2.5_ = 2.5% *w*/*v* medium-viscosity chitosan, C_H1.25_ = 1.25% *w*/*v* high-viscosity chitosan, C_H2.5_ = 2.5% *w*/*v* high-viscosity chitosan, Ag_0.75_ = 0.75% *w*/*v* AgNO_3_ solution, Ag_1_ = 1.0% *w*/*v* AgNO_3_ solution, Ag_2_ = 2.0% *w*/*v* AgNO_3_ solution, PVA = PVA 30–70 kDa and the volume of the components used in each formulation are shown in the brackets.

The activity of Ag^+^ hydrogels will be discussed based on the viscosity of chitosan and concentration of AgNO_3_ because it has electrostatic crosslinking properties that affect the stability of the resultant hydrogels as well as their antimicrobial activity. In Ag^+^ hydrogels, the order of sensitivity also follows the MLC order of Ag^+^ reported in Low *et al.*, 2011 [43], *i.e.*, *C. albicans* > *P. aeruginosa* ≥ *S. aureus* (Figure 5), when the concentration of Ag^+^ is >0.2117% *w*/*v*. However, when the concentration is ≤0.2117% *w*/*v* Ag^+^ (formulation C_L2.5(28)_Ag_2(7)_PVA_10(7)_, C_L2.5(10.5)_Ag_0.75(7)_PVA_10(3.5)_, C_M2.5(10)_Ag_1(5)_PVA_10(6)_, C_M2.5(7)_Ag_1(7)_PVA_10(7)_ and C_H2.5(14)_Ag_2(2)_PVA_10(5)_), the order changes to *P. aeruginosa* > *S. aureus* ≥ *C. albicans*. Experimental results also showed that as Ag^+^ diffuses into the MEA, a precipitate is formed in the agar. This can reduce the bioavailability of Ag^+^ against *C. albicans*, resulting in a shift in the order of sensitivity. Despite its role as a crosslinker, the antimicrobial activity of Ag^+^ is not affected if there are sufficient metal ions available for release from the hydrogel matrix (refer to Figure 5).

In Figure 5A, formulations with >0.2117% *w*/*v* Ag^+^ (C_L1.25(9)_Ag_2(12)_-C_L1.25(8)_Ag_2(13)_) all showed a ZOI against *C. albicans*, unlike formulations C_L2.5(28)_Ag_2(7)_PVA_10(7)_ and C_L2.5(10.5)_Ag_0.75(7)_PVA_10(3.5)_. The observed ZOI was not influenced by the small increase in Ag^+^ concentration from 0.635% to 0.786% *w*/*v*. All formulations showed a ZOI against *P. aeruginosa* and *S. aureus*. The increase in Ag^+^ from C_L1.25(9)_Ag_2(12)_ to C_L1.25(8)_Ag_2(13)_ showed a gradual increase in ZOI, although this increase was not significant (*p* > 0.05). This may be due to the higher free Ag^+^ MLC of *P. aeruginosa* (1.59 × 10^−3^ % *w*/*v*) and *S. aureus* (5.08 × 10^−3^ % *w*/*v*) compared to *C. albicans* (6.35 × 10^−4^ % *w*/*v*) [43]. Medium-viscosity chitosan hydrogels (Figure 5B) showed relatively similar ZOI when compared to those in Figure 5A with comparable amounts of Ag^+^ (C_L2.5(28)_Ag_2(7)_PVA_10(7)_ and C_M2.5(10)_Ag_1(5)_PVA_10(6)_; C_L2.5(10.5)_Ag_0.75(7)_PVA_10(3.5)_ and C_M2.5(7)_Ag_1(7)_PVA_10(7)_). Formulations C_M1.25(22)_Ag_2(20)_ and C_M1.25(10.5)_Ag_2(10.5)_ also showed a similar order of sensitivity as observed in C_L1.25(9)_Ag_2(12)_. The increased viscosity in C_M1.25(22)_Ag_2(20)_ and C_M1.25(10.5)_Ag_2(10.5)_ may increase entrapment of Ag^+^ within the hydrogel, leading to a slight increase in the ZOI against *P. aeruginosa* and *S. aureus*. However, this is not observed in *C. albicans*, as the slower release lowered the availability of Ag^+^, making it difficult to overcome the reaction with MEA and to express antimicrobial activity.

A similar trend applies when hydrogels formulated with high-viscosity chitosan were used, e.g., formulation C_H1.25(14)_Ag_2(10.5)_ in Figure 5C. The higher viscosity of hydrogels in Figure 5C altered the order of sensitivity to *P. aeruginosa* being most sensitive, followed by *C. albicans* and *S. aureus*. Disregarding *C. albicans*, this trend of sensitivity showed similarities to the results obtained from the MLC of free Ag^+^ [43]. In addition to the high viscosity, the interaction between the Ag^+^ and MEA makes it more difficult to monitor the actual extent of antimicrobial activity against *C. albicans*.

**Figure 6:**
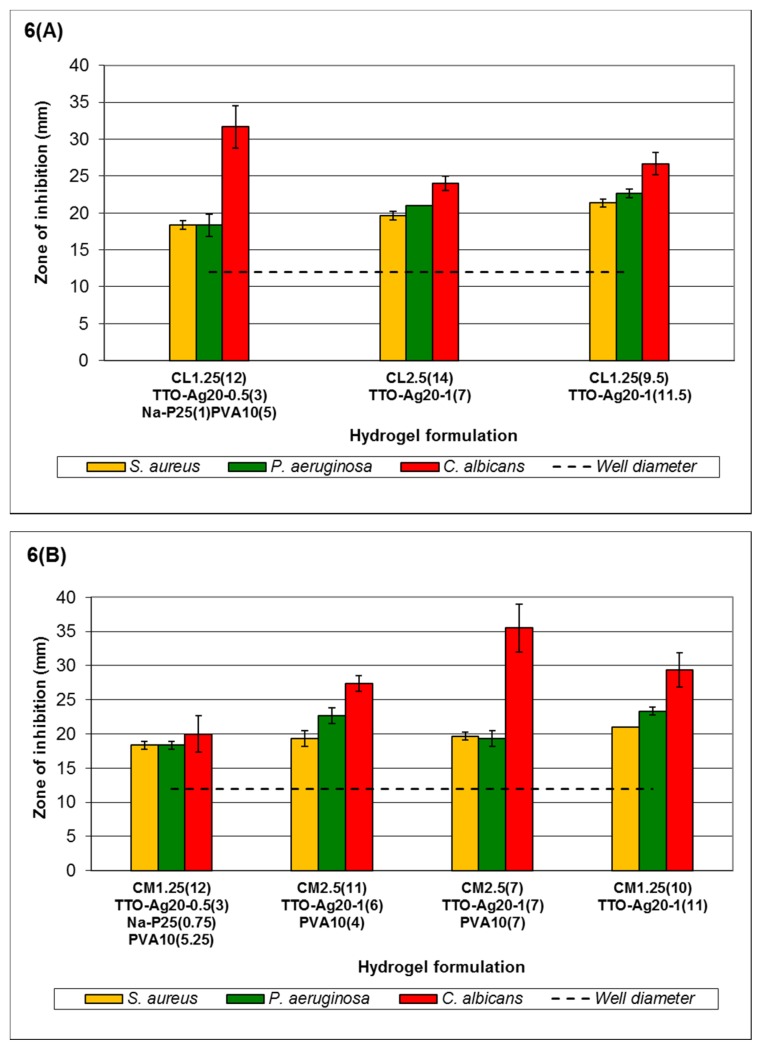

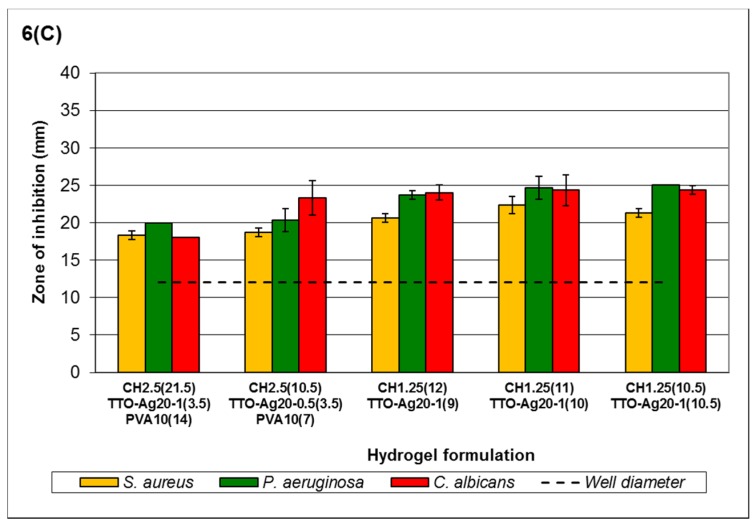
Antimicrobial activity of Ag^+^ + TTO: PVA hydrogels formulated with (**A**) low-viscosity chitosan; (**B**) medium-viscosity chitosan; and (**C**) high-viscosity chitosan. Error bars represent the standard deviation and *n* = 3. In all control experiments (using hydrogels without Ag^+^ + TTO: PVA), there was no observed ZOI (data not shown). Statistical analysis using ANOVA indicated a significant difference in the antimicrobial activity of the formulations against the selected microorganisms (*p* < 0.05). The codes shown in the figure key describe the following: C_L1.25_ = 1.25% *w*/*v* low-viscosity chitosan, C_L2.5_ = 2.5% *w*/*v* low-viscosity chitosan, C_M1.25_ = 1.25% *w*/*v* medium-viscosity chitosan, C_M2.5_ = 2.5% *w*/*v* medium-viscosity chitosan, C_H1.25_ = 1.25% *w*/*v* high-viscosity chitosan, C_H2.5_ = 2.5% *w*/*v* high-viscosity chitosan, TTO-Ag_20-0.5_ = 40% TTO: 0.5% AgNO_3_, TTO-Ag_20-1_ = 40% TTO: 1.0% AgNO_3_, Na-P_25_ = 25% *w*/*v* sodium phosphate, PVA = PVA 30–70 kDa and the volume of the components used in each formulation are shown in the brackets.

According to Figure 6, the order of sensitivity generally follows the MLC response for free agents reported in Low *et al.*, 2011 [43], *i.e.*, *C. albicans* > *P. aeruginosa* > *S. aureus*, for low- and medium-viscosity but not high-viscosity chitosan hydrogels. Formulation C_L1.25(12)_TTO-Ag_20-0.5(3)_Na-P_25(1)_PVA_10(5)_-C_L1.25(9.5)_TTO-Ag_20-1(11.5)_ (Figure 6A) shows that the antimicrobial activity of the hydrogels is dependent on the viscosity and Ag^+^ content in the system. Higher viscosity with increased Ag^+^ forms gels with better stability for entrapment of agents and reduces burst release of agents via erosion. Nevertheless, such properties may allow controlled release to be achieved for sustained antimicrobial activity against less sensitive microorganisms. Small increments in ZOI of the microorganisms were observed as the concentration of agents increased (formulations C_L1.25(12)_TTO-Ag_20-0.5(3)_Na-P_25(1)_PVA_10(5)_-C_L1.25(9.5)_TTO-Ag_20-1(11.5)_, except C_L1.25(12)_TTO-Ag_20-0.5(3)_Na-P_25(1)_PVA_10(5)_ against *C. albicans*). The lower Ag^+^ content in C_L1.25(12)_TTO-Ag_20-0.5(3)_Na-P_25(1)_PVA_10(5)_ may reduce crosslinking to enable a faster release of TTO. Thus, the observed higher ZOI may be due to *C. albicans* having higher sensitivity to TTO. Similarly, this was also observed in the ZOI of formulations C_M2.5(11)_TTO-Ag_20-1(6)_PVA_10(4)_, C_M2.5(7)_TTO-Ag_20-1(7)_PVA_10(7)_ and C_M1.25(10)_TTO-Ag_20-1(11)_ (Figure 6B) against *C. albicans*. The higher concentration of TTO in formulation C_M2.5(7)_TTO-Ag_20-1(7)_PVA_10(7)_ (0.1058% *w*/*v* Ag^+^ and 13.333% *v*/*v* TTO) resulted in the higher *C. albicans* ZOI compared to a relatively similar counterpart, formulation C_M2.5(11)_TTO-Ag_20-1(6)_PVA_10(4)_ (0.1814% *w*/*v* Ag^+^ and 5.7143% *v*/*v* TTO). The ZOI of *S. aureus* and *P. aeruginosa* remained fairly consistent between formulations C_M1.25(12)_TTO-Ag_20-0.5(3)_Na-P_25(0.75)_PVA_10(5.25)_- C_M1.25(10)_TTO-Ag_20-1(11)_.

High-viscosity chitosan hydrogels (Figure 6C) showed slight variation from the MLC data. The order of sensitivity is *P. aeruginosa* ≥ *C. albicans* > *S. aureus*. Similar to all the other formulations, the overall viscosity of the system, the crosslinking capacity of Ag^+^ and the amount of TTO affect the antimicrobial activity of the formulations.

Nevertheless, hydrogels containing both TTO and Ag^+^ managed to maintain their antimicrobial activity despite having agent concentrations lower than those in TTO or Ag^+^ hydrogels. For example, formulation C_H2.5(21.5)_TTO-Ag_20-1(3.5)_PVA_10(14)_, containing 0.0570% *w*/*v* Ag^+^ and 1.795% *v*/*v* TTO, maintained antimicrobial activity when compared to similar formulations (e.g., C_H2.5(14)_Ag_2(2)_PVA_10(5)_ containing 0.0605% *w*/*v* Ag^+^, and C_H2.5(10)_Na-P_50(1)_TTO_35(4)_PVA_10(6)_ containing 6.6667% *v*/*v* TTO). At low concentrations of Ag^+^, formulation C_H2.5(14)_Ag_2(2)_PVA_10(5)_ did not show a clear ZOI against *C. albicans*, while formulation C_H2.5(10)_Na-P_50(1)_TTO_35(4)_PVA_10(6)_ did not show a clear ZOI against *P. aeruginosa*, despite having higher TTO content. The ZOI observed for *S. aureus* remained fairly consistent when comparing the hydrogels formulated with varying chitosan viscosity. The maintenance of activity when using both agents at lower concentrations demonstrates the feasibility of using combined agents at lower effective concentrations.

Incorporation of TTO and/or Ag^+^ into chitosan hydrogels demonstrated the possibility of maintaining antimicrobial activity when using a delivery system. The capacity and performance of antimicrobial agents varies depending on the type of dressing and wound. For the treatment of moist wounds, e.g., ulcers, the absorptive properties of hydrogels make them a better option compared to non-absorptive dressings such as gauzes. Research indicated that there is no single dressing that can produce the optimum microenvironment for all wounds [44], thus the characteristics of formulations and delivery systems need to be tailored to suit the wound site.

Factors such as the differences in physicochemical properties of the hydrogels, including chitosan viscosity, concentration of the crosslinker (Na-P or AgNO_3_) and the amount of available antimicrobial agent (Ag^+^ or TTO), may result in the observed varying order of sensitivity between formulations. Low-, medium- and high-viscosity chitosans derive their rheological properties from the increasing chain length of the polymer. Within each of these groups the concentration of chitosan and the degree of crosslinking also influence viscosity. In addition, increasing the amount of Na-P improves crosslinking and increases hydrogel stability. 

Changes in TTO composition in the hydrogels may occur due to the loss of volatile TTO components during the gel setting period. The availability of Ag^+^ varies according to the concentration required for crosslinking (AgNO_3_ donates the anionic crosslinking nitrate group) and the release of Ag^+^ from the hydrogel. 

Encapsulation of broad spectrum antimicrobial agents (TTO and/or Ag^+^) into hydrogel-based formulations may be a feasible option to enhance biodistribution and biological activity of the agents. Despite the effectiveness of combined treatments, the toxicity of individual agents towards human host cells should also be carefully considered. Although toxicity or irritancy are less likely to occur, various reports of toxicity associated with the misuse/overuse of TTO [12,45], Ag^+^ and silver-containing products have been discussed [10,26,28]. Thus, it is important to find an effective lower combination ratio of TTO and Ag^+^ concentrations to avoid toxicity issues whilst maintaining antimicrobial properties. In this investigation, PVA was used to emulsify TTO to form an oil-in-water emulsion. This will allow more efficient encapsulation into the hydrogels, reducing loss and degradation of volatile components which may affect the oil’s therapeutic activity [37,44]. Emulsifying essential oils can also improve hydrophobic and hydrophilic component distribution due to reduced droplet size [44]. The release kinetics of agents incorporated into chitosan gels may be influenced by factors such as the morphology, size, density, extent of crosslinking, gel formulation ability and swelling of the chitosan-based hydrogel [46].

## 4. Conclusions

The versatility of these agent(s) against a wide range of different microorganisms due to their multiple target sites might be used to improve the current treatment strategies for various chronic wound infections. Combining agents with different intra- and extracellular target sites has two advantages. One is to limit the development of resistance towards these antimicrobial compounds by extending the range of target sites. The activity of microbial agents with a single site of action can much more readily be overcome, thus making it easier for resistance to develop. Another advantage is to reduce the likelihood of toxic effects. 

This study showed the feasibility of developing hydrogels as a controlled release system for the delivery of TTO and Ag^+^ in combination. As a whole, the results from this investigation have provided the basis to develop hydrogel formulations for the delivery of an essential oil (TTO) and metal ion (Ag^+^) as antimicrobial agents for the treatment of acute wounds. The relationship between the variables in the hydrogel formulations and antimicrobial activity requires further work. Consequently, the characteristics of the hydrogel formulations, including the encapsulated agent concentration, can be modified to enhance their potential as a smart delivery system.

## Figures and Tables

**I:** TTO Hydrogels.

Effective Amount of TTO (% *v*/*v*)	Hydrogel Formulation	Vol. of Chitosan (mL)		Vol. of 25% Na-P (mL)	Vol. of 50% Na-P (mL)	35% TTO: 65% PVA_30-70_ (mL)	Vol. of 10% PVA_30-70_ (mL)
		1.25% *w*/*v*	2.50% *w*/*v*				
6.667	C_L2.5(10)_Na-P_50(7)_TTO_35(4)_	-	10.00	-	7.00	4.00	-
6.667	C_L1.25(17)_Na-P_25(17)_TTO_35(8)_	17.00	-	17.00	-	8.00	-
6.667	C_L1.25(8)_Na-P_25(9)_TTO_35(4)_	8.00	-	9.00	-	4.00	-
8.333	C_L2.5(7)_Na-P_25(9)_TTO_35(5)_	-	7.00	9.00	-	5.00	-
6.667	C_M2.5(12.5)_Na-P_50(2.5)_TTO_35(4)_	-	12.50	-	2.50	4.00	-
8.333	C_M2.5(11.75)_Na-P_25(4.25)_TTO_35(5)_	-	11.75	4.25	-	5.00	-
6.667	C_M2.5(12)_Na-P_25(5)_TTO_35(4)_	-	12.00	5.00	-	4.00	-
6.667	C_M1.25(12)_Na-P_50(5)_TTO_35(4)_	12.00	-	-	5.00	4.00	-
6.667	C_H2.5(10)_Na-P_50(1)_TTO_35(4)_PVA_10(6)_	-	10.00	-	1.00	4.00	6.00
6.667	C_H1.25(13)_Na-P_50(4)_TTO_35(4)_	13.00	-	-	4.00	4.00	-
6.667	C_H2.5(22)_Na-P_50(7)_TTO_35(8)_PVA_10(5)_	-	22.00	-	7.00	8.00	5.00
6.667	C_H1.25(10)_Na-P_25(7)_TTO_35(4)_	10.00	-	7.00	-	4.00	-

**II:** Ag^+^ Hydrogels.

Effective Amount of Ag^+^ (% *w*/*v*)	Hydrogel Formulation	Vol. of Chitosan (mL)		Vol. of 0.75% *w*/*v* AgNO_3_ (mL)	Vol. of 1% *w*/*v* AgNO_3_ (mL)	Vol. of 2% *w*/*v* AgNO_3_ (mL)	Vol. of 10% PVA_30-70_ (mL)
		1.25% *w*/*v*	2.50% *w*/*v*				
0.212	C_L2.5(28)_Ag_2(7)_PVA_10(7)_	-	28.0	-	-	7.0	7.0
0.159	C_L2.5(10.5)_Ag_0.75(7)_PVA_10(3.5)_	-	10.5	7.0	-	-	3.5
0.726	C_L1.25(9)_Ag_2(12)_	9.0	-	-	-	12.0	-
0.635	C_L1.25(10.5)_Ag_2(10.5)_	10.5	-	-	-	10.5	-
0.786	C_L1.25(8)_Ag_2(13)_	8.0	-	-	-	13.0	-
0.151	C_M2.5(10)_Ag_1(5)_PVA_10(6)_	-	10.0	-	5.0	-	6.0
0.212	C_M2.5(7)_Ag_1(7)_PVA_10(7)_	-	7.0	-	7.0	-	7.0
0.605	C_M1.25(22)_Ag_2(20)_	22.0	-	-	-	20.0	-
0.635	C_M1.25(10.5)_Ag_2(10.5)_	10.5	-	-	-	10.5	-
0.061	C_H2.5(14)_Ag_2(2)_PVA_10(5)_	-	14.0	-	-	2.0	5.0
0.423	C_H1.25(14)_Ag_2(7)_	14.0	-	-	-	7.0	-
0.635	C_H1.25(14)_Ag_2(10.5)_	14.0	-	-	-	10.5	-

**III:** Ag^+^ + TTO: PVA Hydrogels.

Effective Amount of TTO (% *v*/*v*)	Effective Amount of Ag^+^ (% *w*/*v*)	Hydrogel Formulation	Vol. of Chitosan (mL)		Vol. of 20% TTO: 0.5% AgNO_3_ (mL)	Vol. of 20% TTO: 1% AgNO_3_ (mL)	Vol. of 25% Na-P (mL)	Volume of 10% PVA_(30-70)_ (mL)
			1.25% *w*/*v*	2.50% *w*/*v*				
5.714	0.045	C_L1.25(12)_TTO-Ag_20-0.5(3)_Na-P_25(1)_PVA_10(5)_	12.00	-	3.00	-	1.00	5.00
6.667	0.212	C_L2.5(14)_TTO-Ag_20-1(7)_	-	14.00	-	7.00	-	-
10.950	0.348	C_L1.25(9.5)_TTO-Ag_20-1(11.5)_	9.50	-	-	11.50	-	-
5.714	0.045	C_M1.25(12)_TTO-Ag_20-0.5(3)_Na-P_25(0.75)_PVA_10(5.25)_	12.00	-	3.00	-	0.75	5.25
5.714	0.181	C_M2.5(11)_TTO-Ag_20-1(6)_PVA_10(4)_	-	11.00	-	6.00	-	4.00
13.333	0.106	C_M2.5(7)_TTO-Ag_20-1(7)_PVA_10(7)_	-	7.00	-	7.00	-	7.00
10.480	0.333	C_M1.25(10)_TTO-Ag_20-1(11)_	10.00	-	-	11.00	-	-
1.795	0.057	C_H2.5(21.5)_TTO-Ag_20-1(3.5)_PVA_10(14)_	-	21.50	-	3.50	-	14.00
6.667	0.053	C_H2.5(10.5)_TTO-Ag_20-0.5(3.5)_PVA_10(7)_	-	10.50	3.50	-	-	7.00
8.571	0.272	C_H1.25(12)_TTO-Ag_20-1(9)_	12.00	-	-	9.00	-	-
9.524	0.302	C_H1.25(11)_TTO-Ag_20-1(10)_	11.00	-	-	10.00	-	-
10.000	0.318	C_H1.25(10.5)_TTO-Ag_20-1(10.5)_	10.50	-	-	10.50	-	-

**I:** 1.25% *w*/*v* chitosan low viscosity (Chitosan_L1.25_).

Chitosan_L1.25_	10% PVA (% *w*/*v*)		25% Na-P (% *w*/*v*)	Formulation Code on Graph	Hydrogel Appearance
	13–23 kDa	31–50 kDa			
4	2	-	1	C_L1.25_PVA_13-23_	Overall smooth + soft gel
4	-	2	1	C_L1.25_PVA_31-50_	Overall smooth + soft gel

**II:** 2.5% *w*/*v* chitosan low viscosity (Chitosan_L2.5_).

Chitosan_L2.5_	10% PVA (% *w*/*v*)		25% Na-P (% *w*/*v*)	Formulation Code on Graph	Hydrogel Appearance
	13–23 kDa	31–50 kDa			
4	1	-	1	C_L2.5_PVA_13-23_	Overall smooth + soft gel
4	-	2	1	C_L2.5_PVA_31-50_	Overall smooth + soft gel

**III:** 1.25% *w*/*v* chitosan medium viscosity (Chitosan_M1.25_).

Chitosan_M1.25_	10% PVA (% *w*/*v*)		25% Na-P (% *w*/*v*)	Formulation Code on Graph	Hydrogel Appearance
	13–23 kDa	31–50 kDa			
3	1	-	2	C_M1.25_PVA_13-23_	Rough surface + soft gel
4	-	2	1	C_M1.25_PVA_31-50_	Overall smooth + soft gel

**IV:** 2.5% *w*/*v* chitosan medium viscosity (Chitosan_M2.5_).

Chitosan_M2.5_	10% PVA (% *w*/*v*)		25% Na-P (% *w*/*v*)	Formulation Code on Graph	Hydrogel Appearance
	13–23 kDa	31–50 kDa			
4	2	-	1	C_M2.5_PVA_13-23_	Slightly flakey + soft gel
4	-	1	3	C_M2.5_PVA_31-50_	Rough surface + flakey + dry gel

NOTE: Formulations were combined volumetrically.

**Table 3:** Mathematical modelling of monoterpene release from the hydrogel formulations.

Formulation	Correlation Co-Efficient (*R*^2^)			
	Zero Order	First order	Higuichi	Korsmeyers-Peppas
C_L1.25_PVA_13-23_	0.98	0.72	0.95	0.75
C_L1.25_PVA_31-50_	0.99	0.77	0.94	0.80
C_L2.5_PVA_13-23_	0.98	0.86	0.86	0.90
C_L2.5_PVA_31-50_	0.99	0.76	0.96	0.77
C_M1.25_PVA_13-23_	0.93	0.79	0.98	0.85
C_M1.25_PVA_31-50_	0.98	0.83	0.89	0.91
C_M2.5_PVA_13-23_	>0.99	0.88	0.90	0.92
C_M2.5_PVA_31-50_	0.99	0.83	0.94	0.85
